# High-resolution imaging reveals compartmentalization of mitochondrial protein synthesis in cultured human cells

**DOI:** 10.1073/pnas.2008778118

**Published:** 2021-02-01

**Authors:** Matthew Zorkau, Christin A. Albus, Rolando Berlinguer-Palmini, Zofia M. A. Chrzanowska-Lightowlers, Robert N. Lightowlers

**Affiliations:** ^a^Faculty of Medical Sciences, Newcastle University Biosciences Institute, Newcastle upon Tyne NE2 4HH, United Kingdom;; ^b^Bioimaging Unit, Faculty of Medical Sciences, Newcastle University, Newcastle upon Tyne NE2 4HH, United Kingdom

**Keywords:** human mitochondria, mitoribosomes, click chemistry, protein synthesis

## Abstract

Human mitochondria contain their own genome, mitochondrial DNA, that is expressed in the mitochondrial matrix. This genome encodes 13 vital polypeptides that are components of the multisubunit complexes that couple oxidative phosphorylation (OXPHOS). The inner mitochondrial membrane that houses these complexes comprises the inner boundary membrane that runs parallel to the outer membrane, infoldings that form the cristae membranes, and the cristae junctions that separate the two. It is in these cristae membranes that the OXPHOS complexes have been shown to reside in various species. The majority of the OXPHOS subunits are nuclear-encoded and must therefore be imported from the cytosol through the outer membrane at contact sites with the inner boundary membrane. As the mitochondrially encoded components are also integral members of these complexes, where does protein synthesis occur? As transcription, mRNA processing, maturation, and at least part of the mitoribosome assembly process occur at the nucleoid and the spatially juxtaposed mitochondrial RNA granules, is protein synthesis also performed at the RNA granules close to these entities, or does it occur distal to these sites? We have adapted a click chemistry-based method coupled with stimulated emission depletion nanoscopy to address these questions. We report that, in human cells in culture, within the limits of our methodology, the majority of mitochondrial protein synthesis is detected at the cristae membranes and is spatially separated from the sites of RNA processing and maturation.

## Significance

In mitochondria from various species, the OXPHOS complexes reside mainly in the invaginated cristae membranes, as opposed to the inner boundary membrane (IBM) that parallels the mitochondrial outer membrane. However, the IBM contains dynamic contact sites enriched for translocases that import proteins from the cytosol. As the majority of OXPHOS components are imported and need to be integrated in assembly with the mtDNA-encoded components, where does intramitochondrial translation occur? Here we report: 1) a method for visualizing protein synthesis in human mitochondria at super resolution; 2) that synthesis is enriched at cristae membranes, in preference to the IBM; and 3) that sites of translation are spatially separated from RNA granules where RNA processing, maturation, and mitoribosomal assembly occur.

Following the original observation of microcompartments within the mitochondrion (1, 2), researchers have been driven to investigate the organelle in ever-increasing detail. We have known for many years that mitochondria contain two membranes. More recently, the microarchitecture of the organelle has been investigated following the establishment of fluorescent tools and high-resolution light and electron microscopy or tomography (reviewed in refs. 3–5). It is now clear that the inner mitochondrial membrane (IMM) has highly defined regions. It comprises 1) the inner boundary membrane (IBM) that is closely apposed to the outer mitochondrial membrane (OMM) and where contact sites containing the machinery for importing proteins from the cytosol are found (6, 7); 2) highly invaginated cristae membranes (CMs) that house the majority of the OXPHOS complexes (8–10), in particular the FoF1 ATP synthase, dimers of which help generate the architecture of the membranes (11); and 3) large (10 to 40 nm) complexes termed cristae junctions (CJs) composed of the Mitochondrial contact site and Cristae Organizing System (MICOS) (12–14), which forms the cristae and separates them from the IBM.

There has also been much focus on the mitochondrial genome and how and where it is expressed. This has led to the identity and characterization of the nucleoid (15–18) and, more recently, the mitochondrial RNA granule, a complex formed by phase transition where transcripts are processed and matured (19–21). Translation occurs on membrane-associated mitochondrial ribosomes, which are also at least partially assembled in the RNA granule (22, 23). Exactly where, however, protein synthesis is performed in human mitochondria—whether at the CM, CJ, or IBM, within, close to, or distal to the RNA granules—is unknown. To follow this process in yeast mitochondria, Stoldt et al harnessed an elegant series of experimental approaches (24). The yeast *Saccharomyces cerevisiae* express a battery of translational activators that show specificity to mitochondrial mRNAs (mt-mRNAs), which encode individual components of OXPHOS complexes III, IV, and V (25). Using immunolabeling super-resolution and electron microscopy, data were produced that were consistent with mitochondrial DNA (mtDNA)-encoded complex V subunits being synthesized predominantly on the CMs, while complex III and IV components were synthesized both at the IBM and CM. This approach is not feasible in human cells, as, with the exception of TACO1 (26), human mitochondria do not contain translational activators; moreover, the majority of mtDNA-encoded human proteins are components of complex I, an OXPHOS complex that is absent in *S. cerevisiae*. As translational activators were used as surrogate markers for translation, a direct assay for protein synthesis would now be ideal. One study has utilized a pulse-labeling click-chemistry method to directly measure mitochondrial protein synthesis in intact human cells (27). However, the limited resolution and depth of analysis meant many spatial characteristics of newly synthesized mitochondrial proteins remained undefined. In particular, no information on the submitochondrial localization of protein synthesis in human cells could be obtained.

We report here a comprehensive set of analyses that directly measure spatiotemporal kinetics of mitochondrial protein synthesis. Click chemistry (28) is the technique of choice for this approach, appealing to cell biologists as it utilizes free azide or alkyne labeling moieties that are rarely found in cells. The noncanonical methionine analogs homopropargylglycine (HPG) or azidohomoalanine (AHA) can be substituted for methionine and subsequently visualized in fixed cells with azido or alkyne fluorophores, respectively, in a process referred to as fluorescent noncanonical amino acid tagging, or FUNCAT (29, 30). We have adapted initial protocols (27, 31) and used HPG while inhibiting cytosolic protein synthesis to specifically visualize mitochondrial protein synthesis with both confocal microscopy and super-resolution stimulated emission depletion (STED) nanoscopy. Signals reporting protein synthesis can be detected in various human cell lines after 5 min. Over 90 min of HPG labeling, the proportion of translationally active mitochondrial network (∼50%) remained relatively unchanged. Using this method, we can report that the majority of protein synthesis is first detected at the CMs, colocalizing with mitoribosomal proteins and the translational coactivator TACO1. Further, our STED nanoscopy revealed that signal initiates mainly at sites separated from RNA granules, suggesting either that, if the mitoribosome is loaded with mt-mRNA close to the RNA granule, it must be able to travel to the CM prior to synthesis, or that the mitoribosome is loaded at the CM itself.

## Results

### Measuring Spatiotemporal Kinetics of Mitochondrial Protein Synthesis in Human Cells.

**Fig. 1.**
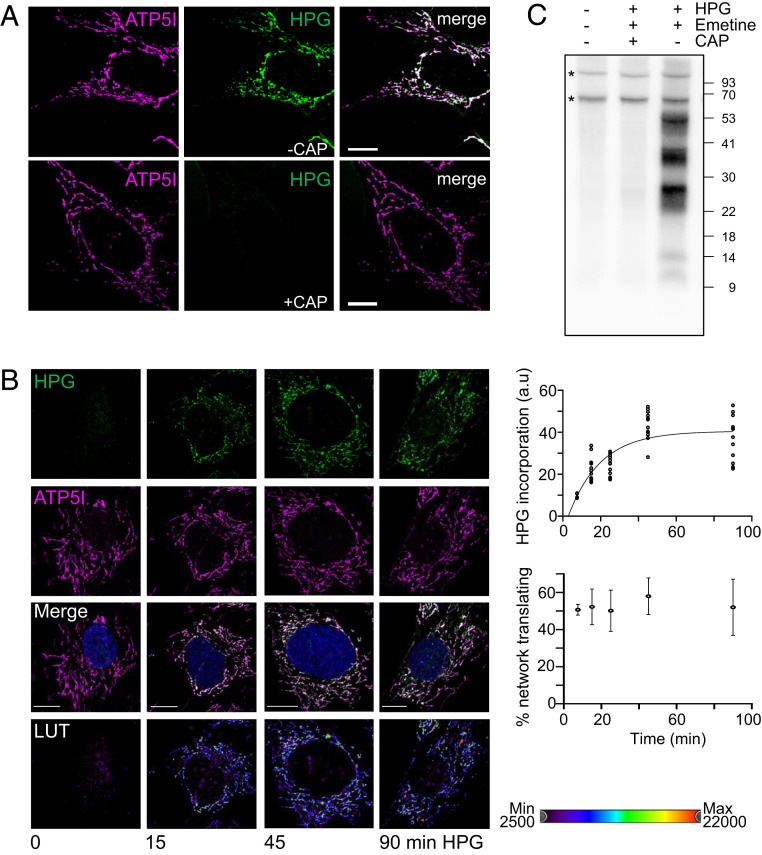
Translation of mtDNA-encoded proteins can be monitored by incorporation of HPG in a time-dependent manner, revealing the distribution across the mitochondrial network. (*A*) U2OS cells were pulsed with HPG (25 min) and cycloheximide in the absence (*Upper*) or presence (*Lower*) of chloramphenicol (CAP). Cells were then fixed, underwent the click reaction, and were stained with antibodies against ATP5I. (*B*) Control fibroblasts were cultured in HPG with inhibition of cytosolic translation by cycloheximide for the time periods indicated. Postfixation, click reactions were performed and samples immunostained as for *A*. All images are representative. (Scale bars: 10 µm.) Relative pixel intensities of HPG are presented as pseudocolor-coded LUTs. The total HPG incorporation as a marker of mitochondria–protein synthesis was plotted over a 90-min time course (*Upper*). The proportion of the mitochondrial network stained with HPG reflecting how much of the reticulum is translationally active was calculated as a percentage at each time point from multiple cells from three independent repeats (*n* = 4, 7.5 min; *n* = 12, 15, 25, 45, 90 min) and is presented graphically (*Lower*). (*C*) Wild-type HEK293 cells were incubated in the presence or absence of inhibitors [emetine and chloramphenicol (CAP)] followed by addition of HPG. Mitochondria were isolated and the click reaction performed as described in *Materials and Methods*. Samples were separated by 12% Bis-Tris PAGE and biotinylated proteins visualized with Streptavidin-HRP and ECL. Asterisks indicate endogenously biotinylated proteins.

Originally, a method had been described to measure newly synthesized cytosolic proteins in intact cells by immunofluorescence. This involved cells in methionine-free media being incubated with the alkyne or azido-methionine derivatives HPG or AHA, respectively. These methionine analogs were incorporated into growing polypeptides, with visualization occurring after fixation and copper-catalyzed cycloaddition of the azido or alkyne fluorophore (29, 32). We adapted this method, termed FUNCAT (fluorescent noncanonical amino acid tagging), to exclusively measure mitochondrial protein synthesis, and further refined previous techniques (*SI Appendix*, Figs. S1–S3) (27, 31). To ensure the method could be widely applied and that observations were robust, all experiments were performed with at least three types of human cultured cells unless otherwise indicated. The cell lines included two transformed lines (HeLa and U2OS) and primary dermal fibroblasts. As can be seen in Fig. 1*A* (U2OS cells) and *SI Appendix*, Fig. S2 (HeLa, fibroblasts), cotreatment of cells with cycloheximide (to inhibit cytosolic protein synthesis) and HPG allowed visualization of a signal that colocalized with the mitochondrial network. The HPG signal was lost upon simultaneous inhibition of cytosolic and mitochondrial protein synthesis (Fig. 1 *A*, *Lower*), demonstrating that the signal exclusively represented HPG-labeled mitochondrially synthesized proteins. Although this demonstrated detectable mitochondrial translation, it did not determine whether HPGylated mt-tRNA^Met^ could be used to initiate synthesis. To address whether the signal represented only elongation of previously initiated proteins, cells were pretreated with puromycin to terminate synthesis prior to addition of HPG. As shown in *SI Appendix*, Fig. S3, mitochondrially derived signal was observed after puromycin pretreatment, consistent with de novo initiation of translation. This indicated that HPGylated mt-tRNA^Met^ could both initiate synthesis as well as contribute to elongation. It is tempting to infer that a subset of HPGylated mt-tRNA^Met^ could have been formylated by mitochondrial methionyl-tRNA formyltransferase, mimicking the endogenous initiation mechanism (33), but recent data suggest that, under certain circumstances, initiation in mitochondria may occur without formylation of an initiating mt-tRNA^Met^ (34).

To determine whether the mitochondrial FUNCAT assay could be utilized to characterize the location and rates of mitochondrial translation, HPG incorporation in the mitochondrial network was measured over a time course (Fig. 1*B*). The rate in fibroblasts was relatively linear over a 45-min pulse, but a clear plateau was evident by 90 min (Fig. 1 *B*, *Upper*), which reflected trends seen in ^35^S-methionine (^35^S-met) radiolabeling of mitochondrial protein synthesis after similar inhibition of cytosolic protein synthesis.

It is not currently known whether translation occurs in discrete foci or is evenly distributed throughout the mitochondrial network. Analysis of fibroblasts revealed that, after a 15-min pulse, a reasonably homogenous pattern of synthesis was present across the network (Fig. 1 *B*, *Lower*). Longer pulses in these and HeLa cells reflected only an increase in pixel intensity [pseudocolored as a lookup table (LUT)] rather than redistribution of HPG signal (Fig. 1). More detailed analysis revealed protein synthesis to be modestly but significantly enriched in all three cell lines tested in perinuclear compared to peripheral mitochondria (*SI Appendix*, Fig. S4).

The established method for visualizing human mitochondrial protein synthesis requires ^35^S-met metabolic labeling (35) and has been adopted by many mitochondrial laboratories over the past 20 y. We attempted to visualize the newly synthesized HPGylated proteins in gel-based systems to avoid the dependency on radiolabel. Following HPG pulse labeling, mitochondria were isolated and HPGylated proteins labeled with picolyl biotin, permitting visualization after transfer. Robust incorporation of HPG into the majority of mitochondrial proteins can be seen (Fig. 1*C*), with a band pattern resembling previous reports for ^35^S-met gels. Labeling was eliminated by chloramphenicol treatment, confirming that the signal represented mitochondrially encoded proteins. However, despite trialing many combinations, our optimized protocol and gel systems were unable to separate and identify all of the 13 species (*SI Appendix*, Fig. S5). In the future, to fully optimize the procedure and resolve all 13 polypeptides, it may be necessary to use gradient gel systems, as recently reported (36), and, to unambiguously assign all species, it will be important to use patient cell lines that carry defined mutations in each of the polypeptides.

### Turnover of Newly Synthesized HPGylated Mitochondrial Proteins.

**Fig. 2.**
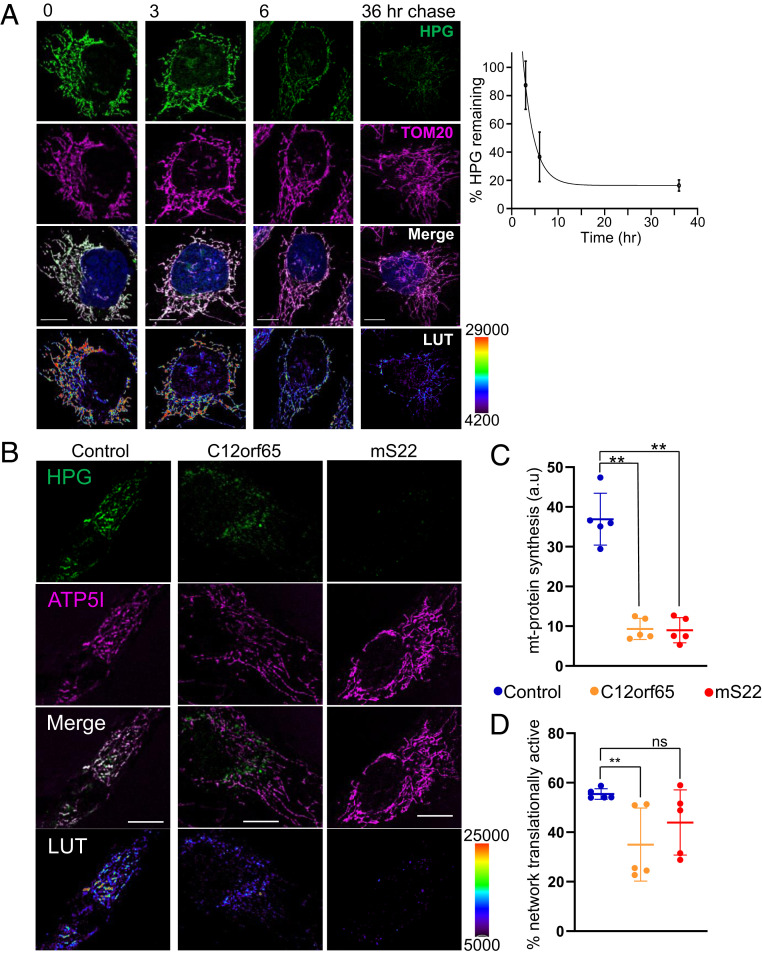
HPG remains detectable in mitochondrial networks after 36 h, and incorporation can reflect translation defects in mitochondrial patient cell lines. (*A*) U2OS cells were pulsed with HPG (green) for 2 h, followed by increasing chase length as indicated. Click reactions were performed as previously described. Mitochondrial outer membranes were visualized with antibodies against TOM20 (magenta), and nuclei were stained with Hoechst (blue). Representative deconvolved confocal images are shown. (Scale bars: 10 µm.) Relative levels of HPG over time are presented as images with pixel intensities as pseudocolor-coded LUTs, and as a graph (*n* = 5 cells per time point). (*B*) Dermal fibroblasts from patients with mutations in nuclear genes (C12orf65 and mS22) known to cause defects in mitochondrial translation were pulsed with HPG (green, 45 min) alongside a control line. The mitochondrial network was visualized with antibodies against ATP5I (magenta). The relative pixel intensity of HPG signal is provided as LUTs. Representative deconvolved confocal images are shown. (Scale bars: 10 µm.) (*C*) Mitochondrial protein synthesis (HPG signal normalized to mitochondrial area; in arbitrary units) was quantified for each cell line [C12orf65 (9.3 ± 2.4) and mS22 (9.0 ± 2.8) HPG relative to control (36.9 ± 5.9)], as was the proportion (as a percentage) of the mitochondrial network where translation was occurring: (*D*) C12orf65 (35.0 ± 13.2%) and mS22 (43.9 ± 11.8%) relative to control (55.4 ± 1.9%). Each data point (*n* = 5) represents analysis of an individual cell, values shown ±SD.

Although it is not immediately relevant to the question of where protein synthesis is occurring in the mitochondrion, we were interested to know whether polypeptides containing multiple HPG molecules were stable. A recent report suggested that, in HeLa cells, the majority of subunits are synthesized in substantial excess and the unassembled components are generally degraded within 3 h, while the fully assembled complexes are highly stable (37). We therefore elected to perform a pulse-chase experiment over 36 h (Fig. 2*A*). Following HPG treatment (2 h), both HPG and the reversible cytosolic protein synthesis inhibitor cycloheximide were removed and methionine added for the chase periods indicated. At 3 h post HPG pulse, ∼87 ± 15% of the signal remained, suggesting that the majority of newly synthesized proteins were not rapidly degraded. Although there was a subsequent decrease after 6 h (36 ± 16%), inferring that a proportion of the newly synthesized mitochondrial protein is not assembled, retention of signal at 36 h (16 ± 4%) was consistent with incorporation of at least a subset of labeled protein into stable complexes.

### Mitochondrial FUNCAT Can Be Used to Identify Mitochondrial Protein Synthesis Defects.

Traditionally, to determine whether individuals have defects in mitochondrial protein synthesis, patient-derived cell lines have been subjected to metabolic labeling with ^35^S-met in the presence of a cytosolic protein synthesis inhibitor (35). One of our goals was to establish mitochondrial FUNCAT as a tool that overcame the need for radiolabel in such assays. To establish whether our methodology could be applied to identify such defects, we used our assay to measure mitochondrial protein synthesis normalized against mitochondrial unit area in control fibroblast and two patient cell lines. Both patients carried defects in mitochondrial protein synthesis: a homozygous exonic deletion [c.210delA, p.(Gly72Alafs*13)] frameshift mutation in the release factor C12orf65 (38) and a mutation in the mitoribosomal subunit mS22. In both cases, a substantially lower signal was noted after 45 min when compared to controls (Fig. 2 *B* and *C*), but there was no such marked variation in the proportion of translationally active network (Fig. 2*D*). Importantly, by 90 min, the difference in signal between patient and control lines was abolished (*SI Appendix*, Fig. S6). This emphasized that kinetics have to be carefully established for each cell line. These data confirm that the mitochondrial FUNCAT assay is suitable for identifying and measuring mitochondrial protein synthesis defects and potentially any changes in distribution of signal within the network.

### Newly Synthesized Protein Is Detected Predominantly at the CMs.

**Fig. 3.**
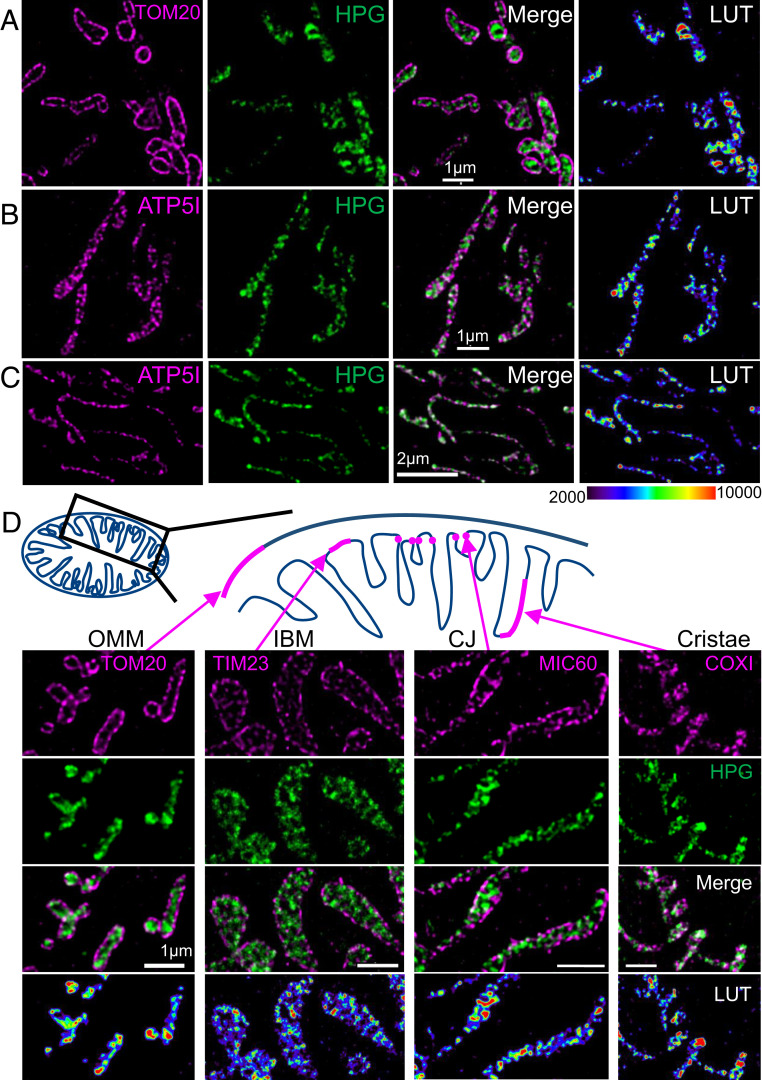
Intramitochondrial protein synthesis is enriched on CMs. De novo protein synthesis was visualized in HeLa cell mitochondria through incubation with HPG (30 min) in the presence of cycloheximide. Cells were stained with antibodies to highlight the positions of the OMMs (TOM20, *A*) or the cristae (ATP5I, *B*). The pseudocolor indicates relative enrichment of HPG incorporation (LUT) reflecting mitochondrial translation. (Scale bars: 1 µm.) (*C*) Control fibroblasts were incubated with HPG (25 min) in the presence of cycloheximide and cells stained with antibodies against ATP5I. (Scale bar: 2 µm.) (*D*) A schematic depicting, in magenta, regions of the OMM, IBM, CJ, and CMs links to relevant panels below of U2OS cells pulsed with HPG (30 min; TIM23, 15 min) in the presence of cycloheximide costained with membrane-specific antibodies and visualized with STED microscopy. (Scale bars: 1 µm.) All representative deconvolved STED images are shown with the relative HPG pixel intensities represented as pseudocolor-coded LUTs.

The main focus of this study was to establish the submitochondrial localization of protein synthesis. To achieve this goal, we adapted the mitochondrial FUNCAT technique to enable visualization by super-resolution STED nanoscopy. One strength of FUNCAT is that fluorophores are attached directly to the methionine analog by the copper-catalyzed alkyne–azide reaction. We were able to optimize the technique using the picolyl Alexa Fluor 594 azide. Colocalization was then determined with various markers using immunofluorescence techniques as described. We first assessed whether newly synthesized proteins could be identified away from the mitochondrial outer membrane (Fig. 3*A*). HPG was pulsed for 30 min, and the signal was compared to that produced from antibodies specific for the outer membrane marker TOM20. High-resolution imaging indicated that newly synthesised proteins are located internal to the outer membrane (Fig. 3 *A* and *B*). Further, the HPG signal clearly shared a location with ATP5I, a component of the FoF1 ATP synthase and member of the CM (Fig. 3 *B* and *C*). Therefore, to assess more precisely where in the IMM the highly hydrophobic mtDNA-encoded proteins were being inserted, we quantified colocalization with markers from the IBM (TIM23, a component of the inner membrane translocase) and CJs (MIC60, a member of the MICOS complex) and a second marker of the CM (COXI, a component of cytochrome *c* oxidase or complex IV). Images in Fig. 3*D* confirm that the majority of HPG signal is found in the CM, colocalizing with COX1, and less so in the IBM. MIC60 was used to visualize the CJ (39), where the IBM begins to invaginate to form the distinct compartment of the CM (Fig. 3*D* and *SI Appendix*, Fig. S7, ATP5l cf. MIC60). Again, the majority of the HPG signal is distinct from and internal to MIC60 puncta, consistent with the newly synthesized proteins being inserted into the CMs. Quantification using Manders’ colocalization coefficient (M2) indicated that, of the HPG signal, 50.8 ± 8.8% (M2 = 0.508) associated with the cristae (COXI), while only 21.5 ± 8.9% colocalized with the IBM marker TIM23 and 12.6 ± 5.4% with CJ marker MIC60. Analysis with Spearman’s rank correlation coefficient also revealed an approximately threefold increase in correlative relationship between HPG and COXI or HPG and ATP5l signals compared to markers for the other submembrane compartments *(**SI Appendix*, Fig. S12), a phenomenon also observed over longer pulses. Similar labeling is also seen when comparing to MIC10, a second member of the MICOS complex and marker of the CJ (*SI Appendix*, Fig. S7). To account for the possibility of movement of newly synthesized proteins within the 30-min pulse, a shorter (7.5-min) pulse was applied that revealed a similar ATPS (complex V beta subunit) distribution (*SI Appendix*, Fig. S8). However, the majority of the data analyzed were after a 15- or 30-min pulse, when the capacity to attain sufficient signal and signal:noise were significantly improved. Taken together, our data support the majority of protein synthesis occurring at CMs.

### HPGylation Is Detected at Sites Proximal to Mitoribosomal Components as Well as Cristae.

**Fig. 4.**
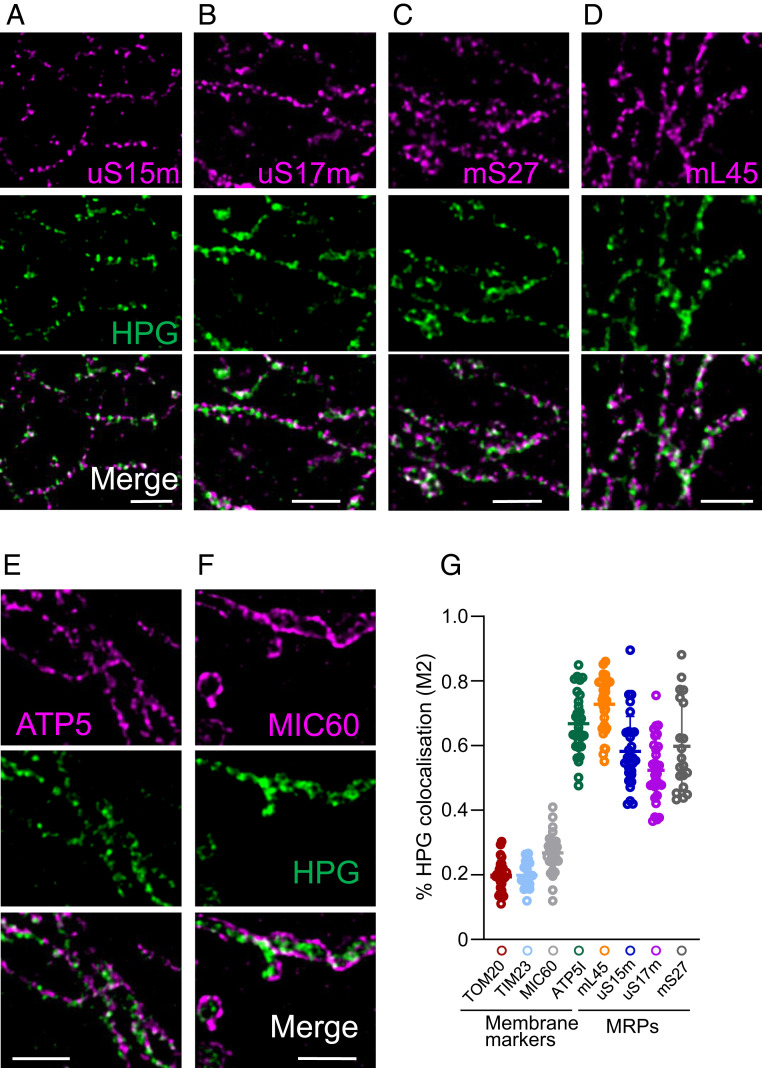
HPG signal colocalizes with mitoribosomal proteins. U2OS cells were pulsed with HPG (30 min in the presence of cycloheximide) prior to fixation and click reactions as described in *Materials and Methods*. Immunostaining was performed with antibodies against mitoribosomal proteins of the small (uS15m, *A*; uS17m, *B*; mS27, *C*) and large subunits (mL45, *D*). To establish whether the HPG and these mitoribosomal components shared the same submitochondrial compartment, markers of the cristae (ATP5I, *E*) and CJ (MIC60, *F*) were also visualized by immunofluorescence. Representative deconvolved STED microscopy images are shown. (Scale bars: 1 µm.) (*G*) Manders (M2) coefficients were derived for % HPG colocalisation with each protein (TIM23 and uS17m, *n* = 20; all others, *n* = 30).

Previous data showed that the majority of HPG signal could first be detected at the CMs. If this signal was a true marker of nascent protein synthesis, colocalization would be expected with markers of components of the mitoribosome, assuming that most of the components were in fully assembled mitoribosomes that were synthesizing protein. This would be consistent with a previous study using immunoelectron microscopy that reported an enrichment of a single yeast mitoribosomal subunit, YmL36, at the CMs (9), an observation consistent with our STED studies. Therefore, to assess the levels of colocalization between HPG signal and the mitoribosomes, we determined the position of three small subunit proteins, uS15m, uS17m, and mS27, and mL45 from the large subunit. As shown and quantified in Fig. 4 and *SI Appendix*, Fig. S9, a 30-min pulse of HPG signal in U2OS cells colocalized strongly with all MRPs measured, and, notably, with the CM marker ATP5I (*SI Appendix*, Fig. S9 *G* and *H*). A much more limited colocalization was seen between HPG and markers of other mitochondrial membrane compartments (*SI Appendix*, Fig. S9 *G* and *H*). Further, a significant increase in colocalization of each MRP was observed with the CM (ATPS) in comparison to the CJ (MIC60) using immunofluorescence alone (*SI Appendix*, Fig. S10).

TACO1 is thought to be a human equivalent of a yeast mitochondrial translational coactivator (26). We therefore reasoned that the intramitochondrial location of TACO1 could be a surrogate marker for nascent synthesis. U2OS lines were generated that inducibly expressed a C-terminal FLAG-tag version of human TACO1. Quantification of Manders’ coefficient revealed that, on induction, 74 ± 8.0% of the TACO1 signal colocalized with HPGylated protein after a 30-min pulse (*SI Appendix*, Fig. S11). A similar correlation was noted with the CM marker ATP5I (63 ± 5.6%), but not with markers of other subcompartments (TOM20, 21 ± 8.5%; MIC60, 19 ± 7.2%). Taken together, these data are consistent with the majority of newly synthesized human mitochondrial protein being inserted into the CMs.

### Mitochondrial Protein Synthesis Colocalizes More Closely With Cristae Than With Mitochondrial RNA Granules.

**Fig. 5.**
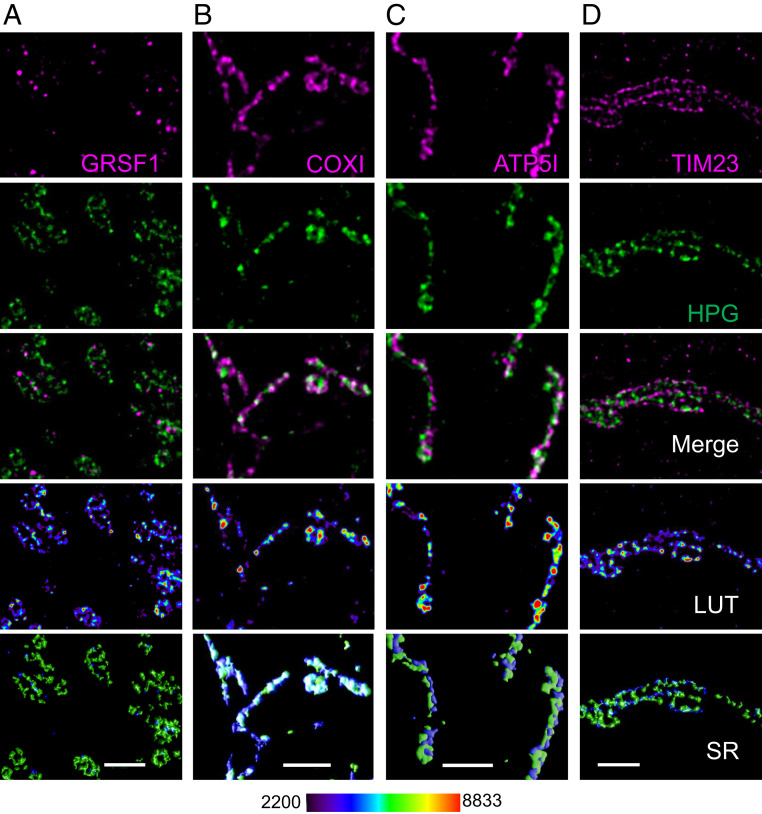
Spatial distribution of mitochondrial RNA granules and intraorganellar translation. U2OS cells were pulsed with HPG (15 min) prior to fixation and click reactions as described in *Materials and Methods*. Immunostaining was performed with antibodies against markers of the mitochondrial RNA granules (GRSF1, *A*), cristae (COXI, *B*; ATP5I, *C*), or IBM (TIM23, *D*). Relative HPG pixel intensities represented as pseudocolor-coded LUTs as well as surface-rendered (SR) representations of the merged data. Deconvolved STED microscopy images are representative of three independent repeats. (Scale bars: 1 µm.)

Mitochondrial gene expression appears highly compartmentalized, with large, juxtaposed complexes (the nucleoid and the RNA granule) in the mitochondrial matrix as highlighted in the Introduction. Is it possible that translation also occurs within the mitochondrial RNA granule, or even in a third juxtaposed structure creating a form of production line to synthesize new proteins? In yeast mitochondria, this idea has been supported by coimmunoprecipitation, proteomics, and super-resolution microscopy studies. Those data revealed an association between the mitoribosomes and other gene expression proteins, leading to the concept of MIOREX, or mitochondrial organization of gene expression (40). To assess whether human mitochondria show a similar arrangement of associated complexes, we used STED nanoscopy to visualize mitochondrion–protein synthesis (HPG) and antibodies to highlight two separate markers of the mitochondrial RNA granule (GRSF1, Fig. 5; and GRSF1 and FASTKD2, *SI Appendix*, Fig. S8). These figures highlight that the majority of HPG signal is spatially distinct from mitochondrial RNA granules. Quantification (Manders’ colocalization analysis) of data from the 15-min pulse-labeled U20S cells revealed that 64.4 ± 8.2% of the HPG signal colocalized with the mitoribosomal subunit uS15m but only 9.5 ± 2.9% with the RNA granule (GRSF1). The HPG signal was also enriched with the cristae marker ATP5l (62.7 ± 2.5%), with a ∼10-fold enrichment compared to GRSF1 (54.7 ± 4.7% vs. 5.4 ± 3.0%) at 30 min (*SI Appendix*, Fig. S12). Even as early as a 7.5-min pulse, the staining profile of HPG more closely resembled ATP5l than FASTKD2 or GRSF1 (*SI Appendix*, Fig. S8, compare white overlap in merge panels of *A* and *B* vs. *C*). Indeed, a similarly low association was also noted between GRSF1 and the translational activator TACO1 (*SI Appendix*, Fig. S11). Taken together, our data show little evidence for compartmentalization of protein synthesis within RNA granules.

## Discussion

We report that mitochondrial FUNCAT is a robust method for visualizing protein synthesis in human cells. This technique represents a significant development in methodology to investigate human mitochondrial protein synthesis, in particular with an adaptation to higher-resolution confocal and STED microscopy. Unlike ^35^S-met radiolabeling that was limited to scintillation counting or gel-based display formats, this approach can visualize spatial information on the distribution of translation within the mitochondrial network. When implemented with super-resolution microscopy and combined with immunofluorescence, as we have here, it allows us to gain a better understanding of how and when different machineries involved in all aspects of gene expression are integrated and how this is affected by different disease conditions. Currently, it is not possible for us to confidently produce consistent data with HPG pulse times shorter than 10 min in all cell types. For this reason, we have exercised caution in our interpretations and conclusions. We believe our data are consistent with the majority of protein synthesis occurring at the CM and distal from the nucleoid or RNA granule. One alternative explanation of our data could be that synthesis occurs at the IBM close to the RNA granule and the new peptides are rapidly moved individually or as partially assembled complexes through the CJs and into the CMs. We believe this is unlikely, as we see no major variation in the distribution irrespective of how long a pulse we use (15 to 120 min), merely an increase in the intensity of the signal. Second, calculation of diffusion coefficients in the CM show, for at least some members of the mature OXPHOS supercomplexes that reside in the CMs, that their diffusion rates are slow (10), reflecting the high protein content in these membranes that makes rapid movement away from the site of synthesis improbable. Third, it has been argued that the CJs make a natural diffusion barrier in the inner membrane, effectively separating the IBM from the CMs (41, 42). Indeed, recent high-resolution studies of live cells have shown that cristae can be biophysically distinct to the IBM as a consequence of CJs providing a form of physical insulation (43). Therefore, it is unclear how easy it would be for complexes made in the IBMs to diffuse through the constricted membranes around the MICOS complexes at the CJ. Finally, we also show a strong colocalization between HPGylated proteins and several markers of the mitoribosome, consistent with the newly synthesized protein remaining in the proximity of the mitoribosomes and not diffusing away. Such a strong spatial correlation was also shown with the single mitochondrial translational activator TACO1. Therefore, our data consistently showed the majority of newly synthesized proteins located internal to OM, IBM, and CJs.

At first sight, our conclusion may seem to contrast with the previous work by Stoldt et al. in yeast, which inferred that mitochondrial subunits of complexes III and IV are made at both inner boundary and CMs dependent on their assembly status, while complex V is preferentially synthesized on CMs (24). However, unlike that investigation that monitored mitochondrial translation via mRNA-specific translational activators and assembly factors, mitochondrial FUNCAT gives a readout of all mitochondrial protein synthesis and cannot distinguish between components of different OXPHOS complexes. It is therefore possible that, as with yeast complex V, human complex I components are largely made at the CM, and, as these constitute the major part of the mitochondrially encoded proteome, this may bias the data, while translation of the complex III, IV, and V components may be more widely distributed across the CM and IBM. Irrespective of this, it is clear that the main signal is visible first at the CM.

A second interesting observation was that synthesis did not appear to be at discrete foci within or apposed to the RNA granule, unlike the situation in yeast (40). Curiously, consistent with our observation are previous data based on quantitative immunoelectron microscopy that reported the majority of the yeast mitoribosomal subunit YmL36 to be associated with the CM, and, notably, when treated with puromycin to terminate synthesis, this selective localization was lost (9). This may also be consistent with further work of Bogenhagen et al. (44) showing that assembly of the human mitoribosome initiates close to the nucleoid or RNA granule but then has late-assembled components potentially added at distal sites. In summary, mitochondrial FUNCAT demonstrates that the majority of mitochondrial protein synthesis occurs at the CM, but currently cannot allow synthesis to be followed in real time. Considering the superb nanoscopy images that are being generated to allow us to visualize the ultrastructure of the mitochondrion in real time (4, 43, 45, 46), we hope to soon adapt our protocol to follow mitochondrial protein synthesis in live cells.

## Materials and Methods

### FUNCAT: Cell Culture and Click-Chemistry Labeling of Mitochondrial Translation in Human Cell Lines.

Cultured dermal human fibroblasts or U2OS or HeLa cells were grown in Dulbecco’s modified Eagle’s medium (Sigma D6429) supplemented with 10% fetal calf serum (Sigma), 1× nonessential amino acids, and 50 µg/mL uridine (Thermo Fisher Scientific) at 37 °C in humidified 5% CO_2_. Access to samples that were excess to diagnostic requirements and were approved for research was covered by the license “Role of mitochondrial abnormalities in disease” (ref. 2002/205) issued by Newcastle and North Tyneside Local Research Ethics Committee. For FUNCAT experiments, cells were first seeded onto glass coverslips and cultured for 1 to 2 d. In vivo labeling of mitochondrial translation products was performed by pulsing cells with methionine-free DMEM containing the methionine analog HPG (Jena Bioscience), while cytosolic translation was inhibited with 50 µg/mL cycloheximide. The TACO1 cDNA spanning the entire open reading frame (from ref. sequence NM_016360.4, nucleotides 157 to 1080) was fused upstream and in frame of the FLAG sequence into pcDNA5/FRT/TO. This, together with pOG44, was used to transfect U2OS Flp-In cells using SuperFect Transfection Reagent (Qiagen), followed by Hygromycin^B^ (100 µg/mL) selection for 14 d. Expression was induced with 1 µg/mL tetracycline. Cells were lysed after 24 h induction, and proteins were separated on a 12% Tris-glycine sodium dodecyl sulfate (SDS) polyacrylamide gel electrophoresis (PAGE) followed by immunoblotting. For immunofluorescence, cells were fixed after 24 h induction.

When inhibiting mitochondrial translation, 100 µg/mL chloramphenicol was also added during the pulse period. If a chase was performed, methionine-free DMEM was replaced with full medium. Cells were prepermeabilized with 0.005% digitonin in mitochondria-protective buffer (MPB; 10 mM Hepes/KOH, 10 mM NaCl, 5 mM MgCl_2_, and 300 mM sucrose in H_2_O, pH 7.5) for 80 s at room temperature to remove unincorporated HPG before being fixed in prewarmed 8% formaldehyde in MPB for 7 min (*SI Appendix*, Figs. S2 and S3). Cells were fully permeabilized with 0.5% (vol/vol) Triton X-100 in PBS (137 mM NaCl, 2.68 mM KCl, and 10 mM Na_2_HPO_4_, pH 7.4) and subsequently blocked with 5% (wt/vol) BSA for 10 min. Pulse-labeled mitochondrial proteins were detected with a copper-catalyzed azide–alkyne cycloaddition [600 μM copper sulfate, 1.2 mM BTTAA, 40 µM picolyl Alexa Fluor 555 (CLK-091-1) or 594 (CLK-1296-1) azide, and 2 mM sodium ascorbate in PBS; all Jena Bioscience] click-chemistry reaction for 40 min. Immunocytochemistry was used to colabel specific mitochondrial targets of interest. This involved incubation with a primary antibody diluted in 5% BSA for 1 h at room temperature, followed by species-specific Alexa Fluor 532 (Thermo Fisher A-11009) or ATTO647N (Sigma M8645) secondary antibodies diluted 1:200 in 5% BSA for 40 min. Nuclei were stained with Hoechst for 5 min, and cells were mounted with ProLong Glass Antifade mountant. Primary antibody details are listed in *SI Appendix*, Table S1.

### Confocal Imaging and Three-Dimensional STED Nanoscopy.

Confocal imaging and three-dimensional (3D) STED nanoscopy were performed on a Leica TCS SP8 gSTED 3× microscope (Leica Microsystems) equipped with white light lasers, HC PL APO 100×/1.40 Oil STED WHITE objective, and 63×/1.40 Oil HC Pl Apo CS2 objective for confocal imaging. A voxel size of (35 to 40) × (35 to 40) × 130 nm (*xyz*) for ×63 confocal and (10 to 20) × (10 to 20) × 100 nm (*xyz*) nm for STED images was used. The fluorophore Alexa Fluor 532 was excited at 527 nm, and STED was performed at 660 nm for super-resolution imaging. Alexa Fluor 555 was excited at 555 nm, and STED was performed at 660 nm. Alexa Fluor 594 was excited at 590 nm and ATTO647N at 646 nm, while STED was performed at 775 nm for both fluorophores. Images were deconvolved and, where indicated, rendered into computer-generated 3D surface maps, using Huygens software (Scientific Volume Imaging).

### Image Analysis.

In each case, unless otherwise stated, 5 to 12 images were analyzed from a single representative experiment that was repeated at least three times with similar results. For determining amount and distribution of mitochondrial protein synthesis within whole-cell confocal images, Columbus software (PerkinElmer) was used on raw images. A mitochondrial translation coefficient was determined by multiplying HPG surface area by mean fluorescence intensity, and this was normalized to mitochondrial surface area (defined by labeling of a general mitochondrial marker such as ATP5I) to give mitochondrial protein synthesis per mitochondrial area. For characterizing other spatial parameters or analyzing STED data, deconvolution and pixel-based colocalization was applied (Scientific Volume Imaging). The colocalization analysis utilized the nonparametric Spearman’s rank correlation coefficient to determine correlation between fluorophore intensities, while Manders’ colocalization coefficients (M1 and M2) were used to calculate proportions of cooccurrence between 0 and 1, which we have described as a percentage with 1 equal to 100%. Where a value for the mean is given followed by a plus/minus value, this represents the SD. To determine any significant differences between populations, the nonparametric Mann–Whitney *U* (Wilcoxon rank-sum) test was performed with a continuity correction where necessary as implemented in the wilcox.test function in R (47).

### Bioorthogonal Noncanonical Amino Acid Tagging: Cell Culture and Click-Chemistry Labeling of Mitochondrial Translation in Human Cell Lines.

Wild-type Hek293 cells were grown in DMEM (Sigma D6429) supplemented with 10% FCS, 1× nonessential amino acids, and 50 µg/mL uridine (37 °C, 5% humidified CO_2_). Medium was refreshed 1 d prior to the experiment, and cells harvested at 50 to 80% confluency. Cells were depleted of methionine by a warm PBS wash and incubated with methionine-free DMEM (20 min) before addition of inhibitors (5 to 10 min) to block either cytosolic (50 µg/mL emetine) or mitochondrial (100 µg/mL chloramphenicol) protein synthesis. HPG was then added (500 µM final concentration) and cells incubated for 2.3 h under normal culture conditions, after which mitochondria were prepared as previously reported (48). Mitochondria were solubilized with 0.4% (wt/vol) SDS at 40 µg protein/60 µL in PBS for 10 min at room temperature, followed by centrifugation (20,000 × *g*, 5 min). The solubilized proteins in the supernatant were used for the click reaction in a final volume of 120 µL (0.2% wt/vol final SDS concentration) with the following components added, in order, to the indicated final concentrations: 20 µM picolyl-biotin-azide (Jena Bioscience CLK-1167-5), 1.2 mM BTTAA (Jena Bioscience, CLK-067-100), 600 µM CuSO_4_, and 5 mM sodium ascorbate. The click reaction was incubated for 60 min at 25 °C and terminated by standard methanol/chloroform protein precipitation. Precipitated proteins were resolubilized in 1× Bis-Tris SDS-PAGE sample buffer and separated on 12% Bis-Tris acrylamide (29:1 Acryl:Bis) gels using 1× MOPS running buffer supplemented with 5 mM sodium bisulfite as a reducing agent. Proteins were transferred onto polyvinylidene fluoride membranes and blocked (5% bovine serum albumen, tween tris-buffered saline) prior to incubation with Streptavidin-HRP and visualization by enhanced chemiluminescence.

## Supplementary Material

Supplementary File

## Data Availability

All study data are included in the article and/or supporting information.
