# Supplementary material for: High-resolution imaging reveals compartmentalization of mitochondrial protein synthesis in cultured human cells
Source: Proc Natl Acad Sci U S A. 2021 Feb 1;118(6):e2008778118. doi: 10.1073/pnas.2008778118 (PMC8017971; doi:10.1073/pnas.2008778118)
Supplement: Supplementary File [file pnas.2008778118.sapp.pdf]

PNAS
www.pnas.org

Supplementary Information for

High resolution imaging reveals compartmentalisation of mitochondrial protein synthesis in cultured human cells.

Matthew Zorkau, Christin A Albus, Rolando Berlinguer-Palmini, Zofia MA Chrzanowska-Lightowlers and Robert N. Lightowlers

Zofia MA Chrzanowska-Lightowlers and Robert N. Lightowlers
Zofia.Chrzanowska-Lightowlers@ncl.ac.uk and
Robert.Lightowlers@ncl.ac.uk

This PDF file includes:

Figures S1 to S12
Table S1

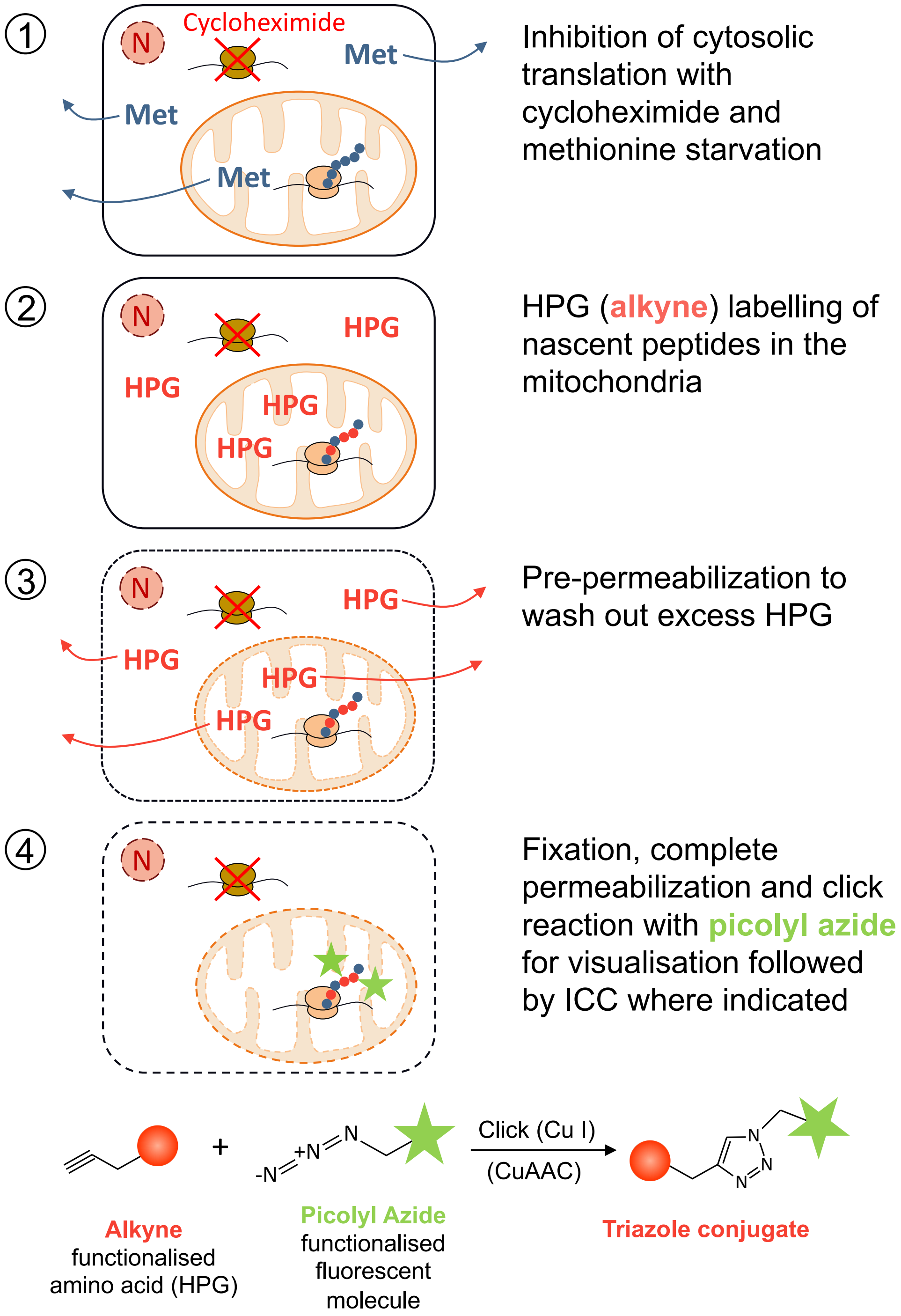

Fig. S1. Schematic overview of the methodology including a simple depiction of the click chemistries involved.
Steps 1 to 4 outline the methods, given in detail in the main text, for HPG labelling of mitochondrial proteins. The copper catalysed alkyne/azide reaction to generate a fluorescent signal is given below panel 4.

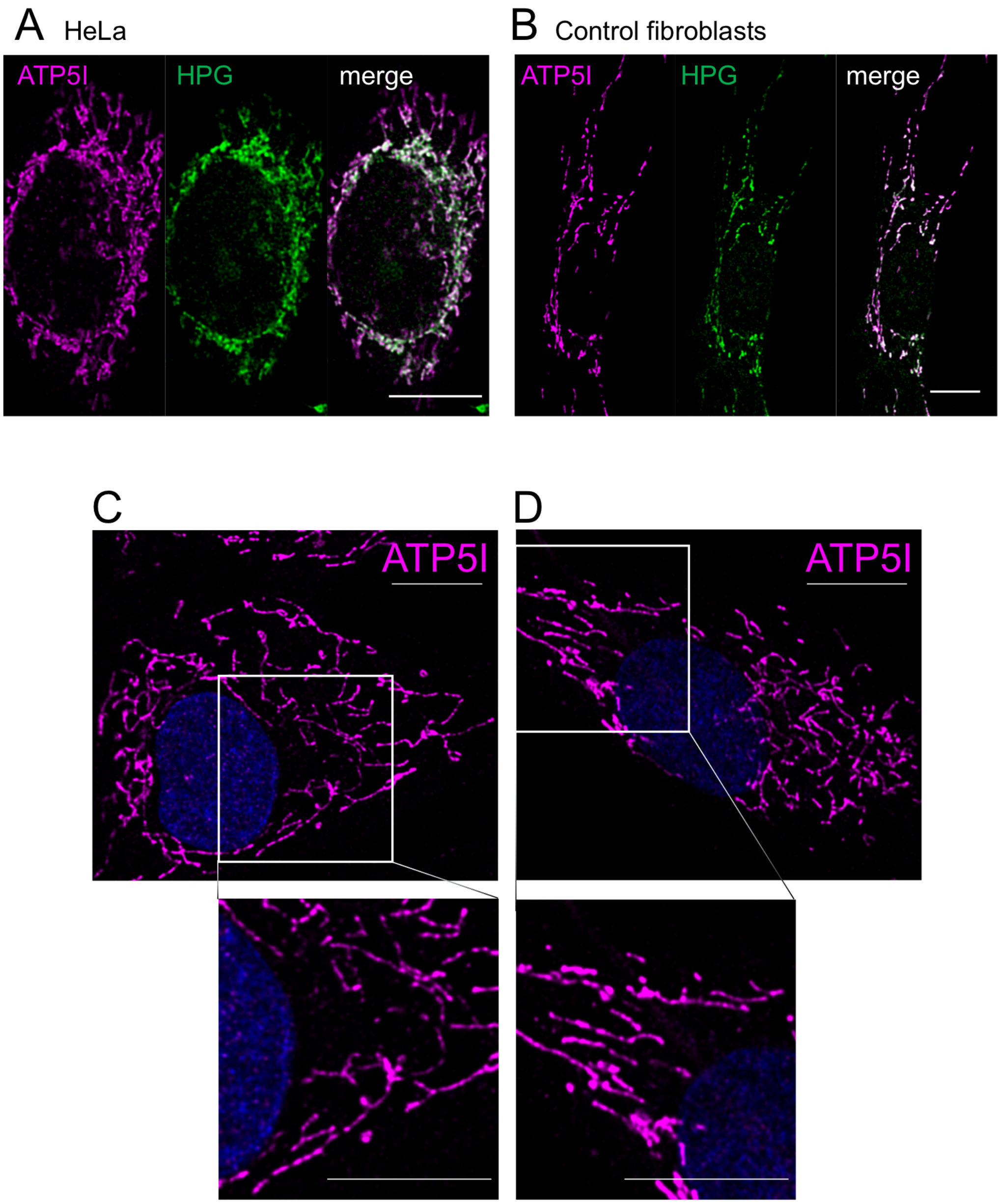

Fig. S2. Mitochondrial protein synthesis can be efficiently visualised within the mitochondrial reticulum in multiple cell types. HeLa cells (A) and control dermal fibroblasts (B) were pulsed with HPG for 25 minutes in the presence of the cytosolic translation inhibitor cycloheximide, followed by the click reaction as described in Methods. Cells were then fixed and stained with antibodies against ATP5I. Representative deconvolved confocal images are shown.

To determine whether the integrity of the mitochondria network was compromised by the FUNCAT procedure, control fibroblasts were left untreated (C), or subjected to an HPG incubation (15 mins, with cycloheximide) and brief digitonin permeabilisation (80 sec) to remove unincorporated HPG (D). Following the standard click reaction (as in Methods) cells were fixed and stained for a marker of mitochondrial cristae (OXPHOS complex V, ATP5I). Representative deconvolved confocal images are shown, panels C and D show nuclei counterstained with Hoechst. Scale bars in panels A-D = 10 µm.

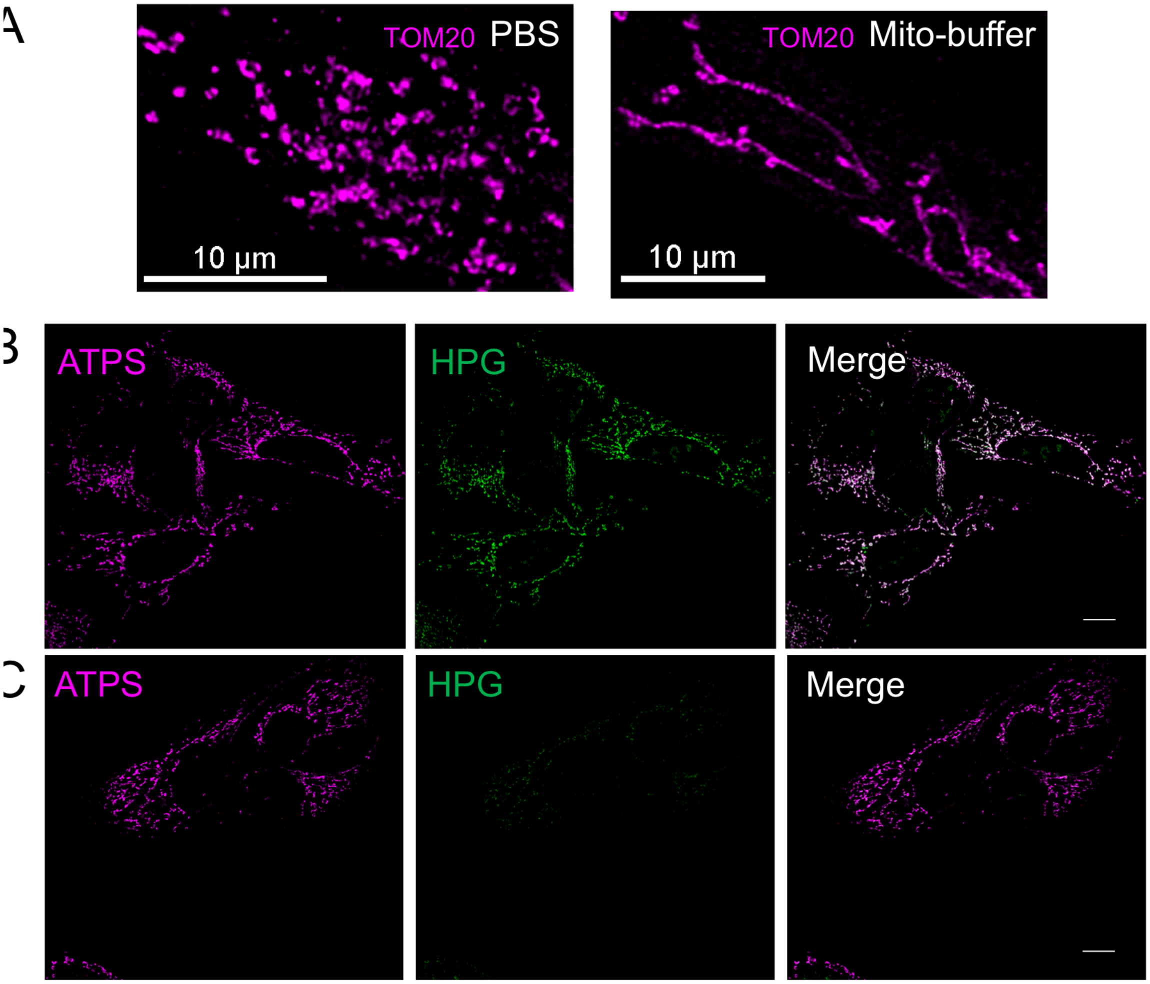

Fig. S3. Optimisation steps to maintain mitochondrial network and to determine whether mitochondrial protein synthesis could initiate *de novo* with HPG as a methionine substitute.
(A) To establish conditions that retained the integrity of the mitochondrial network, control fibroblasts were treated with digitonin (0.005%) for 80 secs in either PBS or mitochondrial protection buffer. Post fixation cells were stained with antibodies against a marker of the outer mitochondrial membrane (TOM20).
Control fibroblasts were pretreated with puromycin (2 hr) to terminate synthesis, prior to addition of HPG. Cells were then incubated with HPG (2 hr) in the absence (B) or continued presence of puromycin (C). Cycloheximide inhibition of cytosolic translation was maintained throughout. Post click reaction and fixation, cells were stained with antibodies against the Complex V protein ATP5B (ATPS) as a marker of the mitochondrial network. Representative deconvolved confocal images are shown with all scale bars = 10 µm.

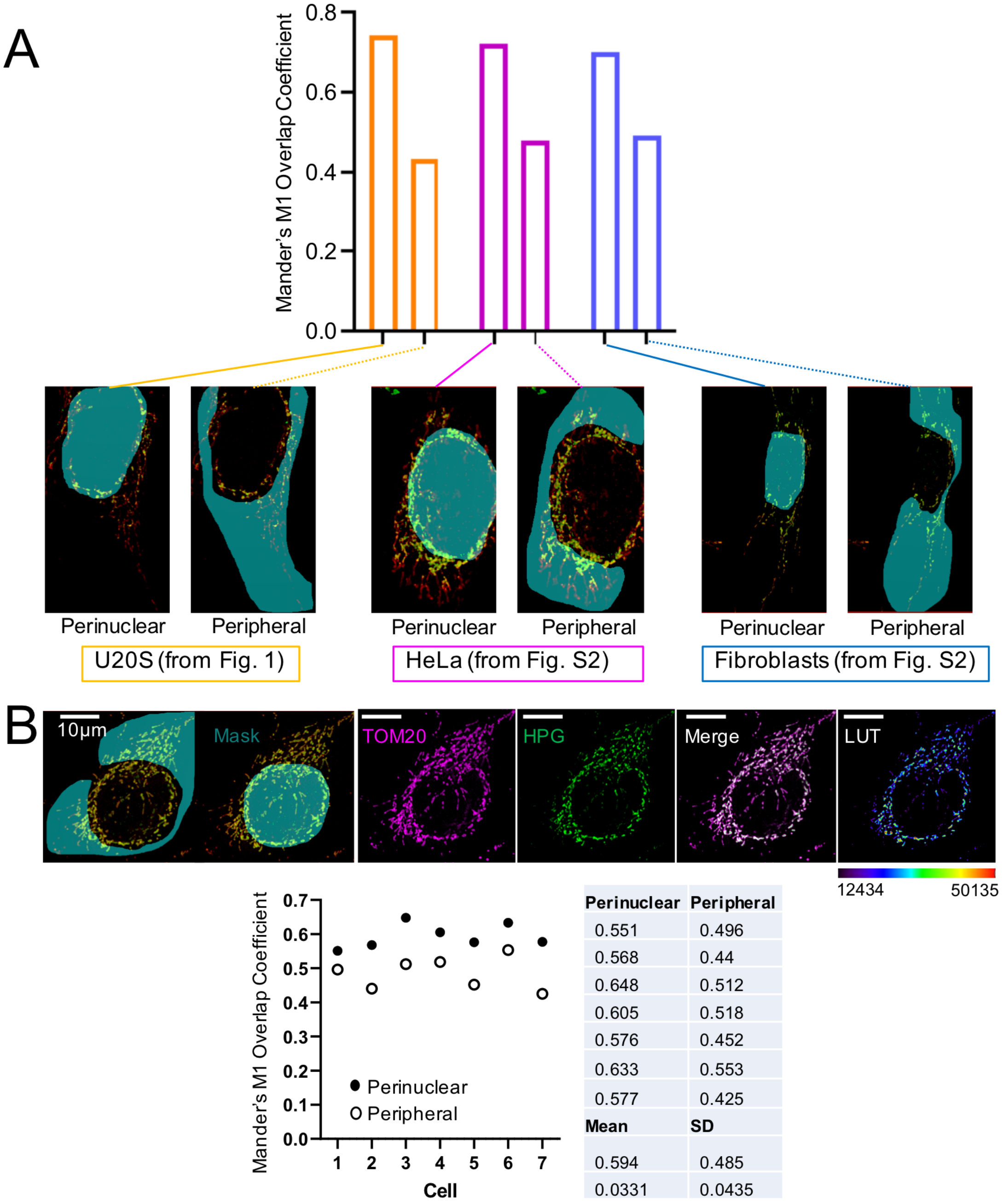

Perinuclear	Peripheral
0.551	0.496
0.568	0.44
0.648	0.512
0.605	0.518
0.576	0.452
0.633	0.553
0.577	0.425
**Mean**	**SD**
0.594	0.485
0.0331	0.0435

Fig. S4. Distribution of mitochondrial protein synthesis between the periphery and the perinuclear region remains similar in different cell types. (A (A) HPG labelling was performed in U2OS, HeLa and fibroblast cells with subsequent immunostaining of the mitochondrial network (ATP5I). Manders' M1 coefficient was used to determine the proportion of the mitochondrial network that was HPG positive in the perinuclear or peripheral mitochondrial network, as masked (turquoise). (B) U20S cells were HPG treated (30 min) and TOM20 labelled before analysis of 7 individual cells in separate fields of view. HPG pixel intensities are presented as pseudo-colour LookUp Tables (LUT). To determine whether the median difference between measures was significant, the two-sided, paired Mann-Whitney test, as implemented in the wilcox.test function in R, was used.

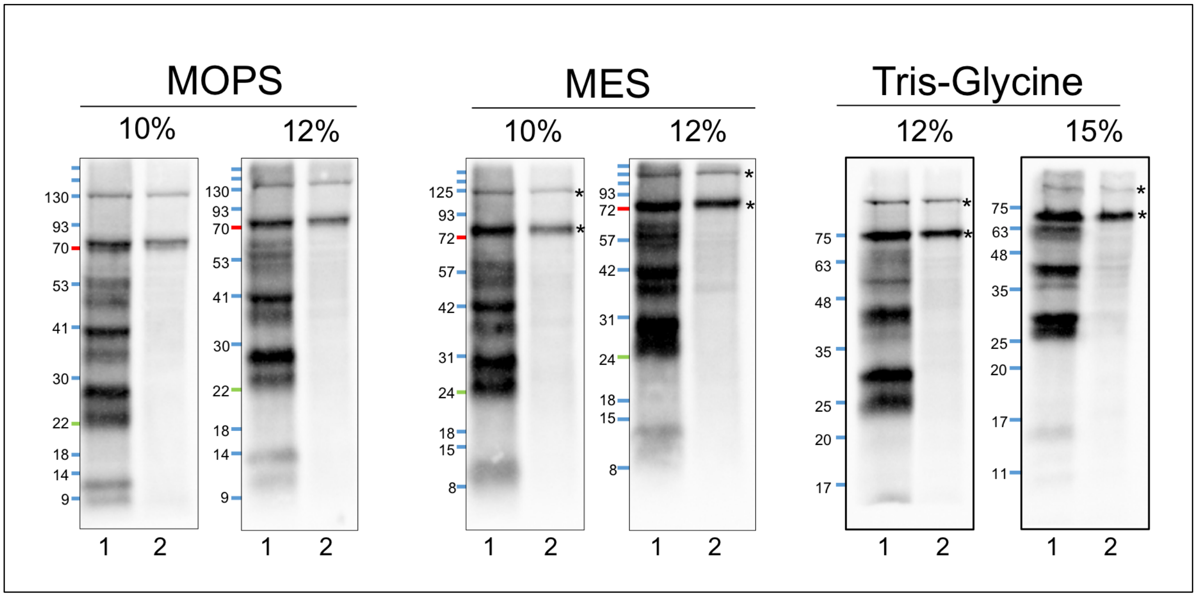

Fig. S5. Different conditions and reagent combinations were tested during optimisation of BONCAT signal of mitochondrial translation
Uninduced Flp-in T-Rex 293 cells carrying a COX8-BioID2 cassette, were incubated with emetine to inhibit cytosolic translation and pulsed with HPG (500 mM, 2 h), in the absence (1), or presence of chloramphenicol (2) as a control that prevents mitochondrial translation. Mitochondria were isolated and samples 1 and 2 were solubilised with SDS (0.2%) and the click reaction performed with p-azide-biotin. Aliquots of samples 1 and 2 were electrophoresed through 6 variants of 29:1 acrylamide/ bisacrylamide gels. MOPS and MES buffers were used with Bis-Tris gels (10%, 12%), and Tris-Glycine running buffer was used with Tris-Glycine gels (12%, 15%). Following wet transfer, membranes were probed with streptavidin-HRP, and signals visualised with ECL. Asterisks indicate endogenously biotinylated proteins. Representative images are shown.

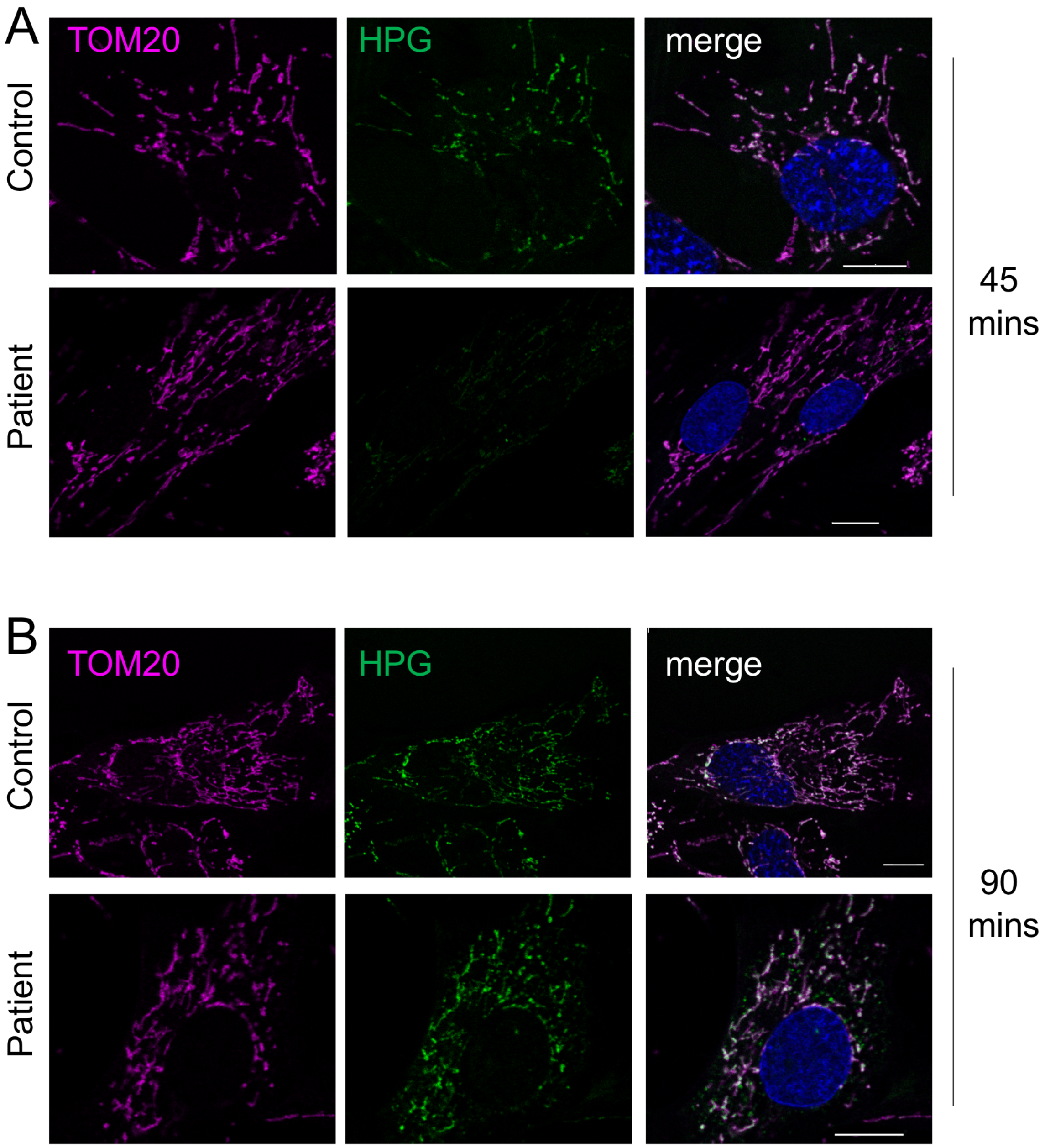

Fig. S6. Mitochondrial protein synthesis defects can be seen in patients with mitochondrial dysfunction. Dermal fibroblasts from control and a patient with a mutation in C12orf65, known to cause a defect in mitochondrial translation, were subjected to 45 (A) and 90 (B) minute HPG pulses. Cells were then fixed and stained with antibodies against an outer membrane marker (TOM20) to highlight the mitochondrial network. Nuclei are stained with Hoechst (blue) in the merged images. Representative confocal images are shown. All scale bars = 10μm.

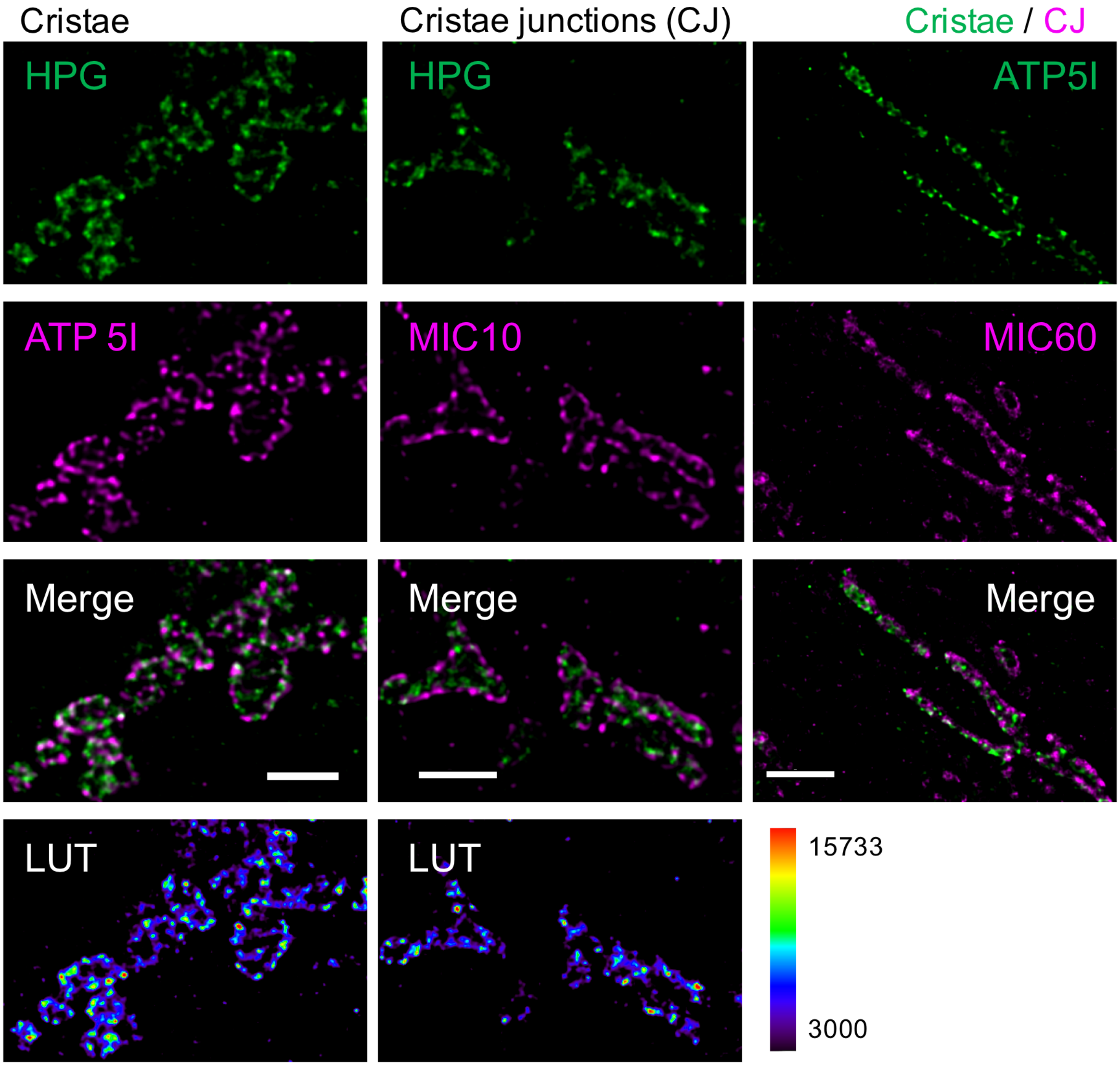

Fig. S7. Mitochondrial protein synthesis occurs at the cristae in preference to cristae junctions. HeLa cells were pulsed with HPG (green) for 30 mins in the presence of cycloheximide followed by click reactions performed as described in Methods. Mitochondrial cristae (left) and cristae junctions (centre) were visualised (magenta) with antibodies against ATP5I and MIC10 respectively. The relative HPG pixel intensities are represented as pseudo-colour coded LookUp Tables (LUT; min 3000, max 15733). To demonstrate the relative positions of cristae and cristae junctions, fixed cells were concurrently stained with antibodies against ATP5I and MIC60 respectively (right). Representative deconvolved super resolution images are shown, all scale bars = 1 µm.

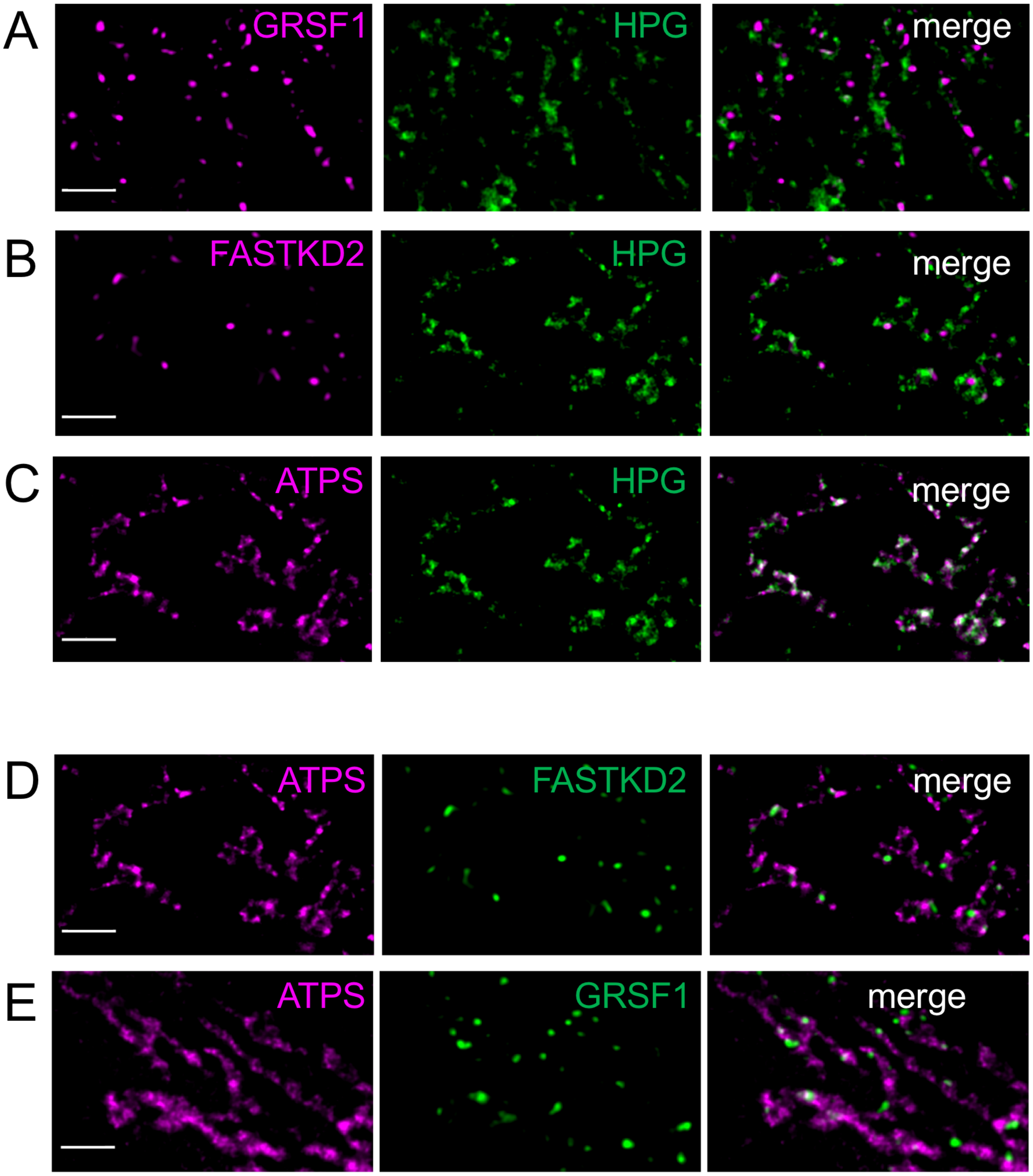

Fig. S8. Mitochondrial protein synthesis colocalises more closely with cristae than with mitochondrial RNA granules. A-C. U2OS were subjected to a short pulse (7.5 min) of HPG (green) in the presence of cycloheximide, followed by immunostaining as described in Methods. To determine the location of mitochondrial RNA granules, two markers were used independently (rabbit antibodies vs GRSF1 (A) or FASTKD2 (B)). To relate these signals and mt-translation to the mitochondrial structure, cristae were also decorated (mouse antibodies against the beta subunit of complex V, ATP5S (C)). Current antibody combinations do not permit FASTKD2/GRSF1 pairing, preventing a combined single integrated image with ATPS and HPG, however, merged images of ATPS with either FASTKD2 (D) or GRSF1 (E) are also shown. These indicate little detectable overlap compared to panel C. Representative super resolution images are shown, all scale bars = 1 μm.

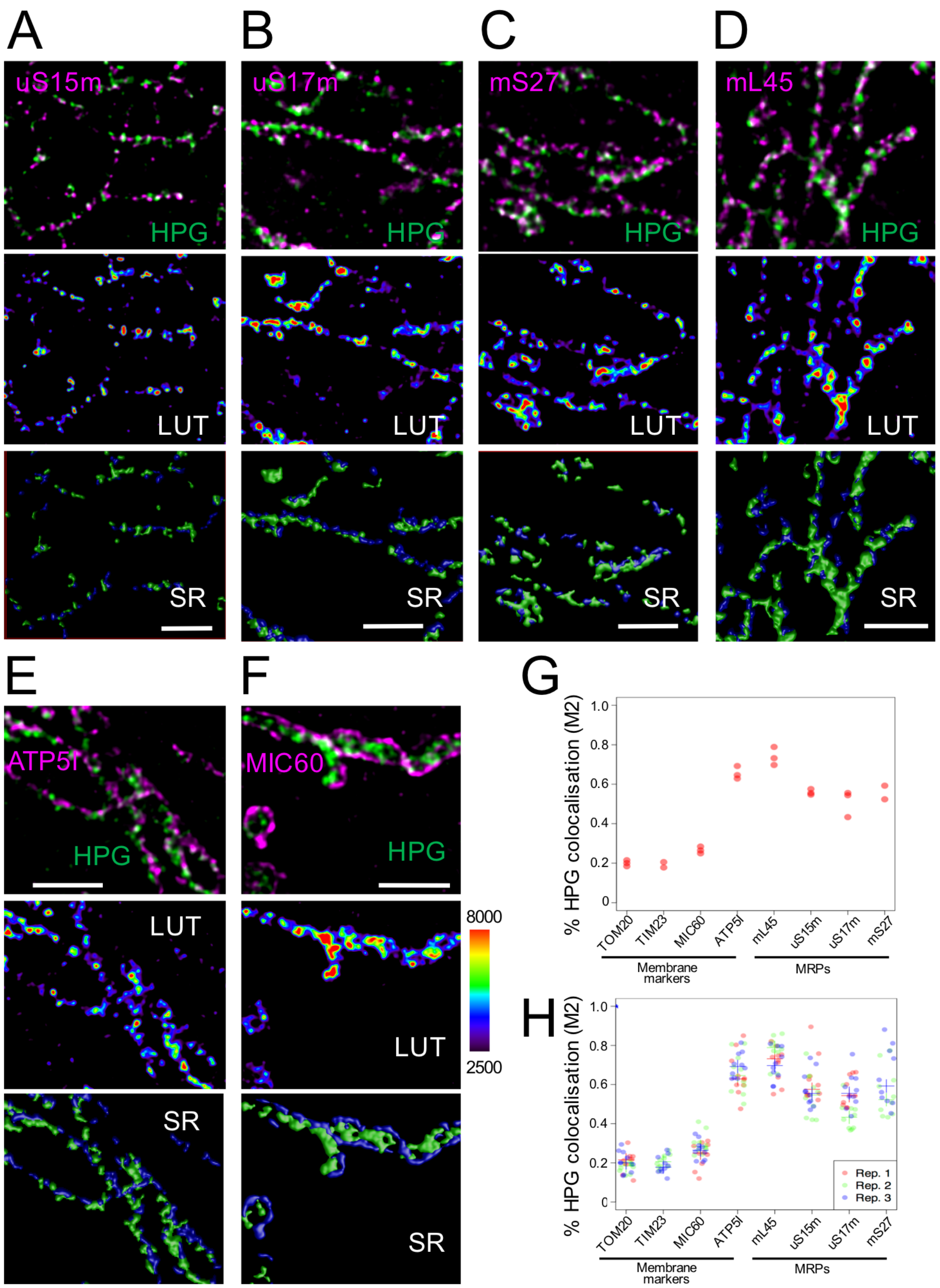

Fig. S9. Co-localisation of HPG signal with mitoribosomal proteins and sub-mitochondrial compartment markers. As described for Figure 4 (main text), U2OS cells were pulsed with HPG (30 min, with cycloheximide) prior to fixation and click reactions with subsequent immunostaining of mitoribosomal proteins (A, uS15m; B, uS17m; C, mS27; D, mL45) and cristae membrane/ cristae junction markers (E, ATP5I; F, MIC60). Representative deconvolved merged STED images from Figure 4 are recapitulated to provide context for relative HPG pixel intensities (pseudo-colour coded LookUp Tables, LUT) and surface rendered (SR) representations. All scale bars = 1 µm. Manders (M2) coefficients were derived for HPG colocalisation with each protein. No significant difference was observed within each set of biological repeats (G) but the colocalisation of HPG with MRPs and ATP5I populations differed significantly (Mann-Whitney test, as implemented in the wilcox.test function in R) from the other markers (H, coloured crosses depict mean/ repeat n = 20 for TIM23/mS27, n = 30 for all others).

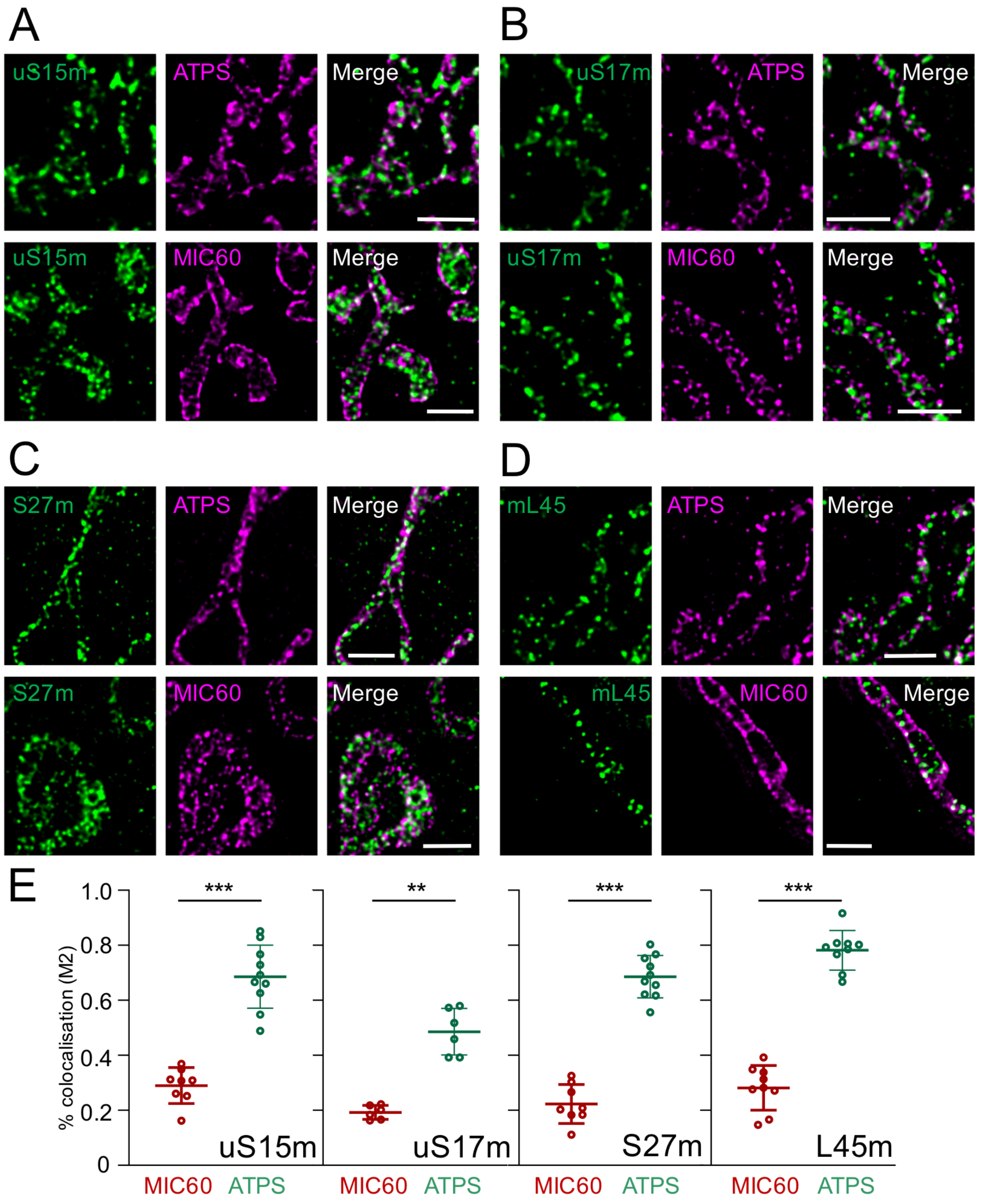

Fig. S10. Distribution of mitoribosomal proteins relative to cristae and cristae junction markers. To determine the relative co-localisation of each of the mitoribosomal proteins uS15m (A, n=8), uS17m (B, n=6), mS27 (C, n=8), and mL45 (D, n=9) with either the cristae (ATPS) or cristae junctions (MIC60), U2OS cells were co-immunostained. Representative deconvolved STED microscopy images are shown and merged images provided. All scale bars = 1 µm. Manders (M2) coefficients were derived for colocalisation of each mitoribosomal protein with ATPS and MIC60 (E). The colocalisation of MRPs with ATPS populations differed significantly (Mann-Whitney test, as implemented in the wilcox.test function in R) from those of MRP/MIC60. Bars designate mean ±SD.

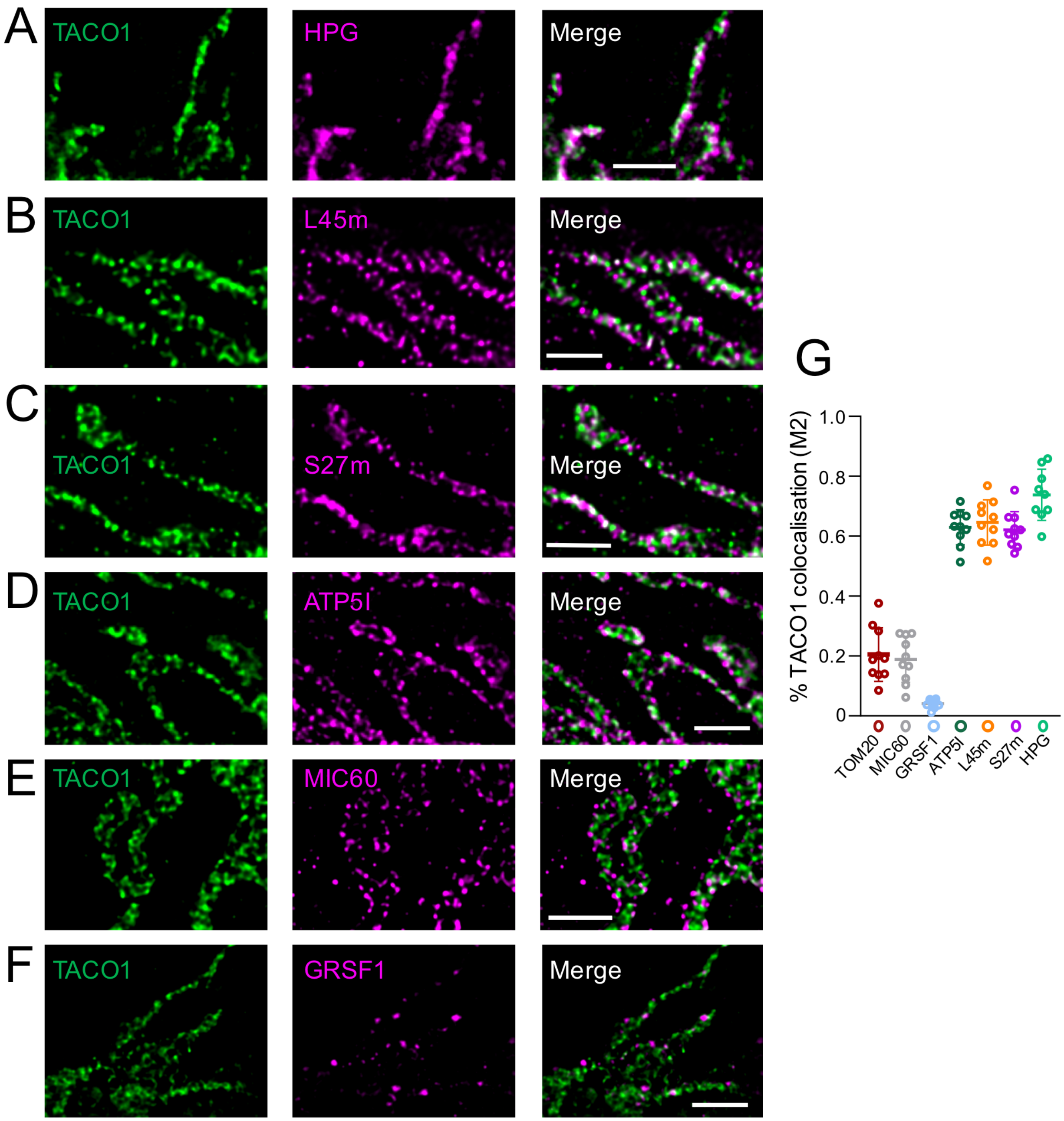

Fig. S11. Distribution of TACO1 relative to mitochondrial markers. To determine the relative co-localisation of the translational activator TACO1 with HPG incorporation (A), mitoribosomal proteins (L45m, B; S27m, C), cristae membranes (ATP5I, D), cristae junctions (MIC60, E) or RNA granules (GRSF1, F), Flp-in U2OS cells stably transfected with TACO1-FLAG were induced (24 hours), fixed and co-immunostained as indicated. HPG n = 9, all others n = 10. Representative deconvolved STED microscopy images and merged images are provided. All scale bars = 1 µm. Manders (M2) coefficients were derived for colocalisation of TACO1 with each of the proteins indicated (G), which indicated that TACO1 colocalisation with TOM20, MIC60 and GRSF1 was significantly different from its colocalisation with HPG and the other proteins indicated (Mann-Whitney test, as implemented in the wilcox.test function in R). Bars designate mean ±SD.

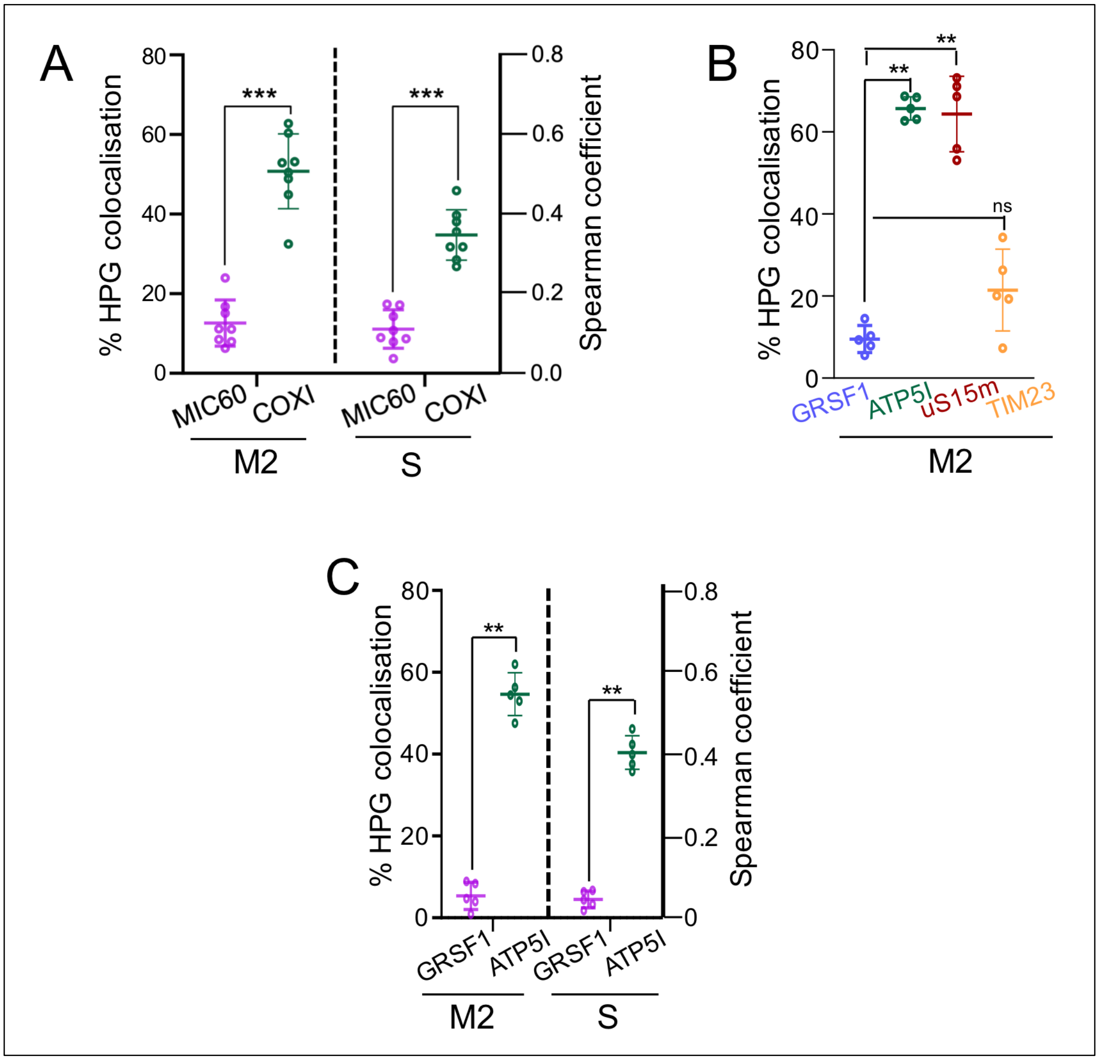

Fig. S12. Mitochondrial protein synthesis colocalisation with various intramitochondrial markers

A. Manders (M2) and Spearman (S) coefficients were derived from data on MIC60 (M2, 12.6 ± 5.4%) and COXI (M2, 50.8 ± 8.8%) presented in Figure 3C, where n=8 cells. The Spearman correlation coefficients for HPG/ MIC60 (0.111 ± 0.045) and HPG/COXI (0.348 ± 0.0529) indicate an approximately 3 fold enrichment of mtDNA encoded protein at the cristae compared to the cristae junction.

B. Manders (M2) coefficients were derived from data on HPG colocalisation with RNA granules (GRSF1; 9.5 ± 2.9%), and markers of the cristae (ATP5I; 62.7 ± 2.5%), mitoribosomes (uS15m; 64.4 ± 8.2%) and the inner boundary membrane (TIM23; 21.5 ± 8.9%) presented in Figure 5 (15min HPG pulse), where n=5 U2OS cells.

C. Manders (M2) and Spearman (S) coefficients were derived from data on GRSF1 and ATP5I after a 30 min HPG pulse. M2 coefficients indicated a significantly higher HPG colocalisation with ATP5I (54.7 ± 4.7%) compared to GRSF1 (5.4 ± 3.0%) n=5. The Spearman correlation coefficients for HPG/GRSF1 (0.045 ± 0.018) and HPG/ATP5I (0.404 ± 0.036) indicate an approximately 10 fold enrichment of mtDNA encoded proteins at the cristae compared to the RNA granules.

In each case significance was determined using the Mann-Whitney test, as implemented in the wilcox.test function in R. Bars designate mean ±SD.

Table S1. Primary antibodies used in this study.

Antigen	Dilution for IF (western)	Host species and isotype	Supplier	Catalogue number
TOM20	1:400	Rabbit IgG	Abcam	ab78547
TIM23	1:200	Rabbit IgG	Protein Tech	11123-1-AP
MIC60	1:400	Mouse IgG1	Abcam	ab110329
MIC60	1:200	Rabbit IgG	Protein Tech	10179-1-AP
MIC10	1:200	Rabbit IgG	Abcam	ab84969
MTCO1	1:200	Mouse IgG2a	Abcam	ab14705
ATP5I	1:200	Rabbit IgG	Protein Tech	16483-1-AP
ATPS	1:200	Mouse IgG1	Abcam	ab14730
FASTKD2	1:200	Rabbit IgG	Protein Tech	17464-1-AP
GRSF1	1:200	Rabbit IgG	Abcam	ab194358
uS15m	1:200	Rabbit IgG	Protein Tech	17006-1-AP
uS17m	1:200	Rabbit IgG	Protein Tech	18881-1-AP
mS27	1:200	Rabbit IgG	Protein Tech	17280-1-AP
mL45	1:200	Rabbit IgG	ThermoFisher	PA5-54778
α-tubulin	1:30000 for western	Mouse IgG1	Abcam	ab7291
FLAG	1:200 (1:1000 for western)	Mouse IgG1 Clone M2	Sigma	F3165
